# Imaging learned fear circuitry in awake mice using fMRI

**DOI:** 10.1111/ejn.12939

**Published:** 2015-06-06

**Authors:** Anjanette P. Harris, Ross J. Lennen, Ian Marshall, Maurits A. Jansen, Cyril R. Pernet, Nichola M. Brydges, Ian C. Duguid, Megan C. Holmes

**Affiliations:** ^1^ BHF Centre for Cardiovascular SciencesQMRIUniversity of EdinburghEdinburghEH16 4TJUK; ^2^ Centre for Cognitive Ageing and Cognitive EpidemiologyQMRIUniversity of EdinburghEdinburghUK; ^3^ Neuroimaging SciencesCentre for Clinical Brain SciencesEdinburghUK; ^4^ Neuroscience and Mental Health Research InstituteCardiff UniversityCardiffUK; ^5^ Integrative PhysicsUniversity of EdinburghEdinburghUK

**Keywords:** amygdala, awake mouse fMRI, cued fear conditioning, nucleus accumbens, Pavlovian conditioning

## Abstract

Functional magnetic resonance imaging (fMRI) of learned behaviour in ‘awake rodents’ provides the opportunity for translational preclinical studies into the influence of pharmacological and genetic manipulations on brain function. fMRI has recently been employed to investigate learned behaviour in awake rats. Here, this methodology is translated to mice, so that future fMRI studies may exploit the vast number of genetically modified mouse lines that are available. One group of mice was conditioned to associate a flashing light (conditioned stimulus, CS) with foot shock (PG; paired group), and another group of mice received foot shock and flashing light explicitly unpaired (UG; unpaired group). The blood oxygen level‐dependent signal (proxy for neuronal activation) in response to the CS was measured 24 h later in awake mice from the PG and UG using fMRI. The amygdala, implicated in fear processing, was activated to a greater degree in the PG than in the UG in response to the CS. Additionally, the nucleus accumbens was activated in the UG in response to the CS. Because the CS signalled an absence of foot shock in the UG, it is possible that this region is involved in processing the safety aspect of the CS. To conclude, the first use of fMRI to visualise brain activation in awake mice that are completing a learned emotional task is reported. This work paves the way for future preclinical fMRI studies to investigate genetic and environmental influences on brain function in transgenic mouse models of disease and aging.

## Introduction

Functional magnetic resonance imaging (fMRI) uses changes in cerebral blood flow to provide a spatially and temporally localised indirect measure of neuronal activation across the whole brain, while a subject performs a cognitive task. Due to the non‐invasive nature of fMRI, this imaging technique has proved invaluable in delineating neural systems underlying sensory processing and higher cognitive and emotional function in the human brain. fMRI is sensitive to changes in brain function due to neuropsychiatric disorders, pharmacological manipulations and genetic differences (Hariri & Weinberger, 2003; Etkin *et al*., 2010), and consequently represents a valuable research and clinical diagnostic tool. fMRI is, however, limited to correlations, and only animal studies in which experimental manipulations of scanned individuals is performed can reveal causal mechanisms. Therefore, fMRI in rodents provides the opportunity for translational preclinical studies into the influence of pharmacological, genetic and environmental manipulations on brain function, which would be difficult to conduct in humans or other animals in which fMRI has recently been used (e.g. pigeons or dogs; Berns *et al*., 2012; De Groof *et al*., 2013).

Typically, fMRI studies in rats and mice have been largely limited to using anaesthetised or resting animals (Jonckers *et al*., 2011), or to studying innate behaviours, such as pup suckling, predator odour exposure or hypoxia (Ferris *et al*., 2005; Duong, 2007; Huang *et al*., 2011), which limits the translatability of this method for clinical impact. In particular, the use of anaesthetics during fMRI keeps the animal still during scanning, but precludes the investigation of brain activity during learned cognitive tasks (such as reward or fear anticipation). Anaesthetics can also suppress the blood oxygen level‐dependent (BOLD) signal, interact with other pharmacological agents or physiological processes (e.g. isoflurane is a vasodilator), and impair comparisons with human fMRI research (Martin *et al*., 2006). Consequently, awake scanning protocols that train the rodent to remain still and relatively calm, without the need for surgical implantation of head‐posts, have been developed and successfully employed to image awake learned behaviour in rats using fMRI. For example, Brydges *et al*. (2013) recently showed fear circuitry activation in response to a conditioned stimulus (CS) that was augmented by early life stress in rats, and Johnson *et al*. (2013) studied brain activation in response to a cocaine‐associated odour cue in awake rats.

To the best of the authors’ knowledge, fMRI has never been used to investigate learned emotional processing in awake mice. Since genetic manipulation of mice is relatively straightforward, there are vast resources of genetically modified mice with neurological defects available. Therefore, fMRI in mice could facilitate preclinical investigation into gene effects and gene‐by‐environment interactions on brain function in models of disease and aging.

Here, for the first time, the feasibility of using fMRI in awake mice is demonstrated. A cued fear learning paradigm was used, in which the neural underpinnings are well characterised, coupled with fMRI to investigate fear circuitry activation in awake mice (LaBar *et al*., 1998; Brydges *et al*., 2013). In particular, recall of a conditioned fear response 24 h after acquisition reveals a key role for the amygdala (Nader *et al*., 2000; Hall *et al*., 2001), therefore recall was investigated 24 h after conditioning. Studies in humans, and the authors’ previous fMRI study in rats, demonstrate unilateral amygdala activation in response to conditioned fear, with activation in the left hemisphere more often than the right (Baas *et al*., 2004; Brydges *et al*., 2013). Additional regions that were predicted to be activated, based on the previous rat study, included the granular insular, hypothalamus and somatosensory cortex, which are involved in processing the fear and anticipation of pain associated with the CS.

## Materials and methods

### Animals

A total of 44 C57bl/6 male mice (34 ± 2 g at the start of the experiment; approx. 12 weeks old, bred in‐house) were used (20 were used in the fMRI experiment, eight were used to test different scanning acclimation protocols and 16 were used in a separate behavioural experiment to confirm acquisition of the conditioned response). All mice were housed in small groups (four–six per cage) with *ad libitum* access to water and standard chow, in a humidity‐ (50–60%), temperature‐ (21 °C) and light‐ (on 07:00–19:00 h) controlled environment. Prior to scanning, all mice were handled daily for 5 days (5–10 min/day) to minimise handling stress. All animal experiments were approved by the University of Edinburgh Ethical Review Committee, and studies were carried out in strict accordance with the UK Home Office Animals (Scientific Procedures) Act 1986 and the Council Directive 2010/63EU of the European Parliament and the council of 22 September 2010 on the protection of animals used for scientific purposes.

### Determining protocol to acclimate mice to the MRI scanning environment

**Figure 1:**
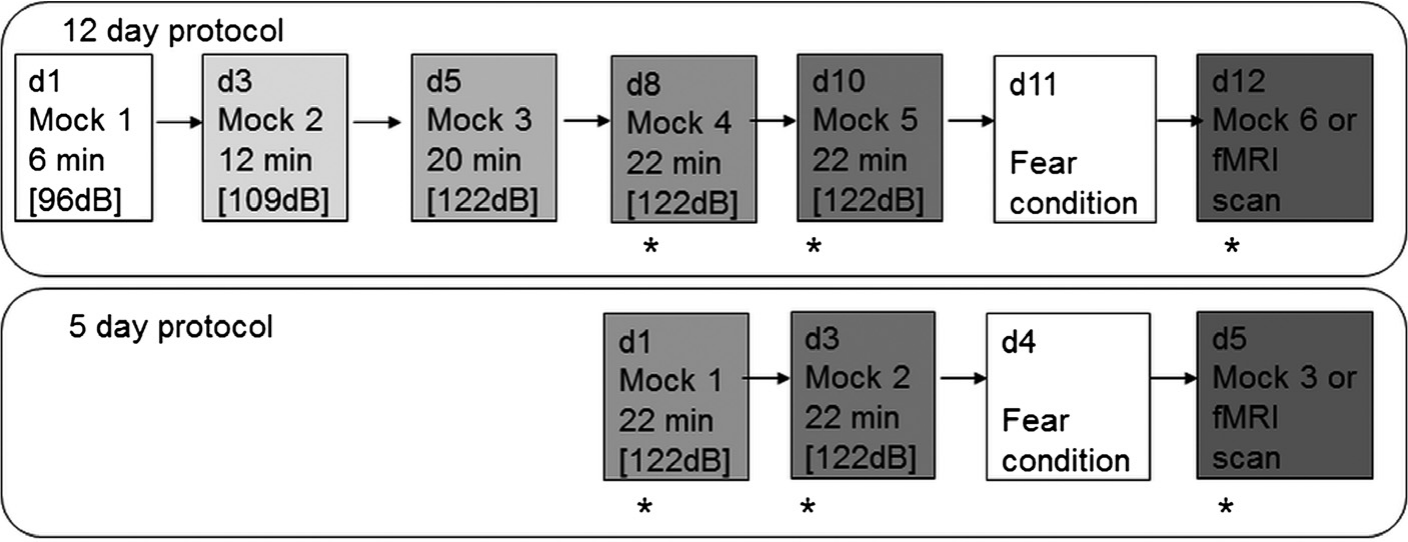
Experimental timeline for the 12‐ and 5‐day protocols. Mice were acclimatised to the scanning environment by simultaneously exposing them to the magnetic resonance imaging (MRI) restraint apparatus and MRI pulse sequence noise (sound level in dB) in ‘mock scans’ over 12 or 5 days. The 12‐day protocol was used for the final functional (f)MRI experiment. Mice underwent cued fear conditioning on Day 11, and the blood oxygen level‐dependent (BOLD) signal was measured in the MRI scanner during retrieval of the conditioned response 24 h later. *Indicates when physiological parameters were measured.

To acclimate mice to the scanning environment, mice were exposed to the restraint apparatus (Animal Imaging Research LLC, USA) and ‘pulse sequence sounds’ in ‘mock scans’ in a custom‐built mock scanner. To determine an optimal protocol, a 5‐day protocol in one cohort of mice (*n* = 4) was compared with a 12‐day protocol that introduced the mice gradually to the pulse sequence sound and restraint. The 5‐day protocol follows that described in Brydges *et al*. (2013), and is as follows: on Days 1, 3 and 5, mice were placed into a mock scanner for 22 min with 122 dB full‐volume pulse sequence (Fig. 1). The 12‐day protocol was as follows: on Day 1, mice were placed in a mock scanner for 6 min with the scanning sequence at 96 dB (approximately 26 dB below actual scanner volume). On Day 3, mice were placed in the mock scanner for 12 min with sound level at 109 dB (13 dB below scanner), and on Day 5 for 20 min with sound level at 122 dB (i.e. full volume). The mice were then mock scanned for 22 min with full volume on Days 8, 10 and 12 (Fig. 1). During the final three mock scans, respiration rate, heart rate, number of body movements (determined by visual assessment, e.g. leg kicks, arched back) and body weight were measured. Corticosterone levels were measured in blood samples that were taken immediately following the mock scans between 12:00 and 17:00 h (measured by tail nick using an in‐house radio‐immunoassay as previously described: Harris *et al*., 2013). To compare the two protocols, data from the final mock scan only were analysed by unpaired *t*‐test, *P *< 0.05 was deemed significant.

### Procedure for ‘mock’ and fMRI scanning

Mice were lightly anaesthetised (1–2% isoflurane in air; 50 : 50 at 1 L/min) for < 4 min whilst placed in and out of the MRI restraint apparatus (Animal Imaging Research, LLC, MA, USA). All limbs were lightly secured with surgical tape, and the trunk of the body was gently held within a cylindrical body restrainer, a plastic shoulder bar slotted over the mouse and into the plastic cylindrical restraint tube to further prevent excess body motion. The mouse's incisors were secured over a bite bar that was used to place the head into position inside a nose cone that delivered anaesthetic. A foam headband was placed over the ears to reduce noise exposure and to hold the head securely in position. The mouse, in the body restraint tube, was then slotted inside the cylindrical chassis of the quadrature volume coil, which clamped the foam headband gently in place. Respiration was constantly monitored (during set up and scanning) by means of a pressure transducer placed under the torso (MR‐compatible Small Animal Monitoring and Gating System, SA Instruments), and a rectal thermistor probe was inserted to monitor and maintain body temperature at 37 ± 0.5 °C by means of a feedback‐controlled warm air system (MR‐compatible Small Animal Heating System, SA Instruments). The mouse in the restraint apparatus was then placed in the bore of the mock or MRI scanner as appropriate.

The 12‐day mock protocol produced better acclimatisation (see results below), therefore this acclimatisation procedure was used for the fMRI experiment.

### The cued fear conditioning paradigm

The day after the last mock scan (Day 10; Fig. 1), mice underwent cued fear conditioning (Day 11; Fig. 1), and fMRI was performed 24 h later on Day 12. Mice in the paired group (PG, *n* = 12) were placed in the conditioning chamber (30 × 25 × 32 cm; Coulbourn Instruments, PA, USA), and over the course of a 25‐min period were exposed to five pairings (every 5 ± 1 min) of the CS: 10 s of high‐intensity rapidly flashing blue LED light (5 Hz max intensity flashes, 50/50 duty cycle) that co‐terminated with the unconditioned stimulus (US): a 2‐s, 0.5‐mA foot shock delivered through the grid floor. Freezing was measured manually, directly and continuously throughout each inter‐shock interval. The percentage of time spent freezing during the inter‐shock interval showed a steady increase from the pre‐shock interval (0% time freezing) until the third inter‐shock interval (after the third foot shock) where freezing plateaued at 71 ± 12% (± SD). Mice in the unpaired group (UG, *n* = 8) were conditioned with the same parameters as PG mice, with the exception that the CS was explicitly unpaired with the US during conditioning.

Behavioural confirmation of the learned association between the CS and US was carried out in a subset of animals that did not undergo fMRI scanning (PG, *n* = 8; UG, *n* = 8). Mice were conditioned and then tested for freezing behaviour in response to presentation of the CS 24 h later in a retrieval trial, which was conducted in a novel context (grey Perspex open top box, dimensions 60 × 60 cm and 30 cm high). In the novel context, after 2 min the CS was presented (flashing light identical to training) for 2 min followed by 2 min rest (no light), this was repeated a total of three times. The percentage of time spent freezing was measured manually, directly and continuously throughout each 2‐min CS and rest period. A 2 group × 2 conditions repeated‐measures (RM) anova was conducted to test if the PG showed higher conditioning than the UG.

### fMRI image acquisition parameters and task paradigm

Imaging was carried out using a 7T MRI scanner (Agilent Technologies, Yarnton, UK) equipped with a high‐performance gradient set (120 mm inner diameter, maximum gradient strength 400 mT/m), using a 36‐mm quadrature volume coil (Animal Imaging Research, LLC, MA, USA) for radio frequency transmission and reception. A structural image of each mouse was acquired under anaesthesia (1–2% isoflurane in oxygen/air, 50/50, 1 L/min) using a fast spin echo sequence with repetition time = 3000 ms; echo train length = 8; effective echo time = 36 ms; 20 slices; slice thickness = 0.8 mm; axial orientation, field of view = 19.2 × 19.2 mm; matrix = 192 × 192 (in‐plane resolution 100 μm); eight signal averages.

Following acquisition of the structural image, animals were allowed to wake up before beginning the functional scanning paradigm. Functional scanning began once the mouse's respiration rate reached > 100 breaths/min, which typically took 8 ± 3 min. While isoflurane clears from the blood in 7–10 min, effects of this anaesthetic could be present in the brain 30 min following withdrawal (Thrane *et al*., 2012); however, effects would be similar in the PG and UG. After a 5‐min baseline period, the CS was presented in the scanner using a custom‐built array of high‐intensity blue LEDs. The test paradigm consisted of 2‐min blocks of flashing light (CS) alternating with 2‐min rest blocks (during which the CS was not presented), this was repeated a total of three times (without any shocks being administered) with a randomised starting order (total of three blocks of CS and three blocks of rest).

Functional image acquisition used a fast spin echo sequence with repetition time = 2500 ms; echo train length = 16; effective echo time = 37.22 ms; slice thickness = 0.8 mm; axial orientation, field of view = 19.2 × 19.2 mm; matrix = 64 × 64 (in‐plane resolution 300 μm). Functional scanning was run for a total of 103 volumes (17 min 10 s); each volume took 10 s to acquire and consisted of 16 slices covering from the anterior olfactory area to the beginning of the cerebellum (approx. bregma 2.96 mm to −5.68 mm in the Franklin & Paxinos, 2008 atlas). A sample of the raw functional images can be seen in Fig. 3B.

### Image preprocessing

Preprocessing and analysis was performed using statistical parametric map (SPM)8 (http://www.fil.ion.ucl.ac.uk/spm/software/spm8/). Firstly, to facilitate analysis in SPM, all images were scaled up by a factor of 10 in the *x*,* y* and *z* dimensions to account for the relative size difference between human and rodent brains. Then for each mouse the time course of functional images was realigned to remove minor motion, and a mean functional volume was generated using 4th degree B‐spline interpolation. The structural image was then co‐registered to the corresponding mean functional image using affine registration to maximise mutual information. Segmentation of the structural image, using tissue probability maps for grey and white matter and cerebrospinal fluid, was then combined with spatial normalisation to a template (Sawiak *et al*., 2012) using mutual information affine registration with medium bias regularisation and a resampled voxel size of 1 × 1 × 1 mm to match the template (grey matter, white matter, cerebrospinal fluid: Sawiak *et al*., 2009), and the same normalisation parameters were applied to the functional images. All realigned and normalised images were then smoothed using an isotropic Gaussian filter kernel with full width at half maximum 6 mm^3^ (equivalent of 0.6 mm^3^ in unscaled data) to increase signal‐to‐noise, and allow for small anatomical and functional variations between animals (Mikl *et al*., 2008).

### Assessment of motion during scanning

Head motion (displacement) was calculated using the translation and rotation parameters that were generated from the rigid body correction of head motion during the preprocessing realignment step (Van Dijk *et al*., 2012). Mean translation and mean rotation of the brain between volumes was estimated from the translation parameters in the *x* (left/right), *y* (anterior/posterior) and *z* (superior/inferior) directions. The mean displacements in 3D space for each brain volume were calculated as the root‐mean‐square of the translation parameters [displacement = square root (*x* ^2^ + *y* ^2^ + *z* ^2^)/10] and expressed in mm. Rotation was a single angle measurement based on Euler's rotation theorem that expresses any 3D rotation as a single angle and corresponding axis of rotation. Rotation was calculated as the average of the absolute value of the Euler angle of the rotation between adjacent volumes using:

cos[cos(phi)cos(theta) + cos(phi)cos(psi) + cos(theta)cos(psi)  + sin(phi)sin (psi)sin(theta)−1]/2,

then divide by 10 to account for the multiplication by 10 during the preprocessing, where phi, theta and psi are the rotational parameters around the three axes (*x*,* y*,* z*; Van Dijk *et al*., 2012).

### Correlation of displacement with signal‐to‐noise ratio (SNR)

To determine if motion during fMRI explained the signal in the amygdala in the PG and the UG, and to determine if there were differences between the groups in the SNR; the SNR was calculated per image using 3 voxels in the amygdala and 3 voxels outside the brain for each of the 100 scan volumes and for each mouse. The SNR was then correlated with the displacement (in mm) between each of the 100 scan volumes, and the resulting Pearson's *r* ^2^ were converted to Fisher's *Z*‐scores, and two‐sample *t*‐tests between groups were then performed to compare PG and UG and determine if motion related to SNR.

### Image analysis

First‐level analysis (intra‐subject) was performed on each mouse using a general linear model in SPM8. The three CS presentations were modelled as a single regressor, and the 5‐min baseline period and rest periods were modelled as an implicit baseline in the model. It was predicted that the amygdala may show extinction of activation across the three CS presentations, and so parametric modulation was used to model a linear decrease from first CS to the third CS to investigate any extinction of neuronal response to the CS. All data were modelled by a boxcar convolved with the SPM8 canonical haemodynamic response function (HRF). Movement parameters generated in the realignment step were added to the model as multiple regressors. Event‐dependent high‐pass filtering was used whereby the cutoff period was 580 s. One contrast image per mouse was entered into a second‐level random effects model (inter‐subject), within‐group effects were examined using one‐sample *t*‐test and between‐group effects were examined using a two‐sample *t*‐test.

Group‐level SPMs were considered significant at *P *< 0.05 corrected for multiple comparisons using cluster‐level correction based on random field theory (*P*_FWE_; FWE, family‐wise error). SPMs are presented overlaid onto an average structural image (created using the average of all normalised structural images) with voxel‐wise threshold *P*_uncorrected_ **< 0.001 (unless otherwise stated), and the number of voxels within the cluster; peak *T‐*value and coordinates in *x*,* y* and *z* dimensions of the peak voxel within the cluster are reported.

Our previous fMRI studies in rats and other human fMRI studies demonstrate that the left amygdala is activated more often than the right in response to fear (Baas *et al*., 2004); therefore, a small volume correction (SVC) was applied (with *P*_uncorrected_ **< 0.001 voxel‐wise threshold) using an anatomically defined left amygdala mask (see below) to test the *a priori* determined hypothesis that the left amygdala would show greater activation in the PG than in the UG in response to the CS (clusters with cluster‐level *P*_FWE_ **< 0.05 were considered significant).

To explicitly investigate laterality of amygdala activation across the three CS presentations, a second general linear model was used to model the three CSs as separate regressors and the rest periods as an implicit baseline. This enabled parameter estimates to be extracted from the left and right amygdala during the first, second and third CS presentations for the PG and UG. Anatomically defined masks covering anterior to posterior basolateral, central and basomedial nuclei were created using MRIcro (Rorden & Brett, 2000) using the Franklin and Paxinos atlas as a guide. MarsBaR Region of Interest toolbox for SPM (Brett *et al*., 2002) was then used to extract and export the parameter estimates for statistical analysis with JMP software using RM anova, group (PG or UG) was a main effect, and CS presentation (CS1, CS2, CS3) and hemisphere (left or right) were repeated measures. To formally test for lateralisation of amygdala activation, left and right parameter estimates from the PG were analysed by paired *t*‐test, *P *< 0.05 was deemed significant.

## Results

### Twelve‐day protocol is optimal to acclimate mice to the scanning environment

The behavioural and physiological parameters that were recorded during the final mock scan for both protocols are presented in Table 1.

**Table 1:** Physiological parameters (± SD) during the final mock scan in the 5‐ and 12‐day protocols

Parameter	12‐day protocol. In final mock:	5‐day protocol. In final mock:
Corticosterone (nmol/L)	191 ± 88	527 ± 163**
Body weight loss (g)	−0.8 ± 0.3	−2.1 ± 0.4**
Body movements	5 ± 3	18 ± 12***
Respiration (per min)	206 ± 60	253 ± 26

Body weight and corticosterone levels were measured after the mock scan, *n* = 3–4/parameter. **P < 0.01; ***P < 0.001; unpaired *t*‐test between both protocols.

During the final mock scan, mice that experienced the 5‐day protocol had higher levels of corticosterone [+336.3 nmol/L; 95% confidence interval (CI) (109, 564); *t*_6_ = 3.6, *P* = 0.01], lost more weight [−1.4 g; 95% CI (−2, −0.6), *t*_5_ = 4.5, *P* = 0.006] and had higher levels of movement [+13 moves; 95% CI (6, 21), *t*_14_ = 4.0, *P* = 0.001] than did mice that experienced the 12‐day protocol. These results were taken as an indication that the 12‐day protocol generated better acclimation than the 5‐day protocol.

### Motion during fMRI

**Figure 2:**
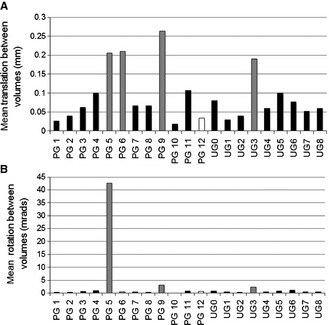
Assessment of movement during functional magnetic resonance imaging (fMRI). (A) Mean translation (mm). (B) Mean rotation (mradians) for each mouse from the paired group (PG) and unpaired group (UG) are shown. Functional voxel dimensions were 0.3 × 0.3 with slice thickness 0.8 mm, therefore mice with movement > 0.15 mm were removed from the analysis. Mouse ‘PG5’ was excluded due to excessive rotation and translation, and mice ‘PG6’, ‘PG9’ and ‘UG3’ were excluded due to excessive translation (> 0.15 mm; grey bars). Mouse ‘PG12’ was removed from analysis due to low respiration rate during scanning (max* *< 150 breaths/min; white bar).

**Figure 3:**
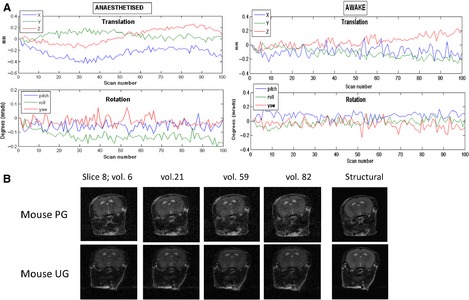
Movement during functional magnetic resonance imaging (fMRI). (A) Movement parameters derived from the realignment step for one anaesthetised and one awake mouse for scaled data. Translation is in mm and represents movement in the *x*,* y* and *z* planes of the scanner, and rotation is in mradians. (B) Raw functional images prior to all preprocessing steps from two representative mice (one from the paired group, PG, and one from the unpaired group, UG). Slice 8 from four different volumes is shown, plus the corresponding structural slice.

Assessment of motion for each mouse during the fMRI session is presented in Fig. 2. From 20 mice, five mice were excluded from the final analysis (one for excessive translation, three for excessive rotation and translation depicted as grey bars in Fig. 2, and one mouse had low respiration during scanning, depicted as white bar in Fig. 2). In Fig. 3A, the movement parameters, derived from the realignment step in the analysis, of two example mice (anaesthetised and awake) during the functional scanning protocol are shown. Although small rotations and translations are seen when the animal is awake, these never exceeded half a voxel in mice included in the analysis (voxel size: 0.3 × 0.3 × 0.8 mm). A representative sample of the raw functional and structural images from two mice showing the same slice from different volumes is shown in Fig. 3B to demonstrate quality of the raw images.

There was no significant difference in the SNR/motion correlations between the PG and UG groups (mean *r* ^2^ value, PG: 0.016 ± 0.01; UG: 0.02 ± 0.01; *t*_13_ = 0.32, *P* = 0.75), and the average SNR across the functional scans in the amygdala did not significantly differ between the PG and UG (mean SNR, PG: 7.6 ± 0.4; UG: 8.0 ± 0.2; *t*_13_ = 0.77, *P* = 0.45), confirming that movement does not explain our signal in the amygdala.

### Behavioural confirmation of learned fear in response to the CS

**Figure 4:**
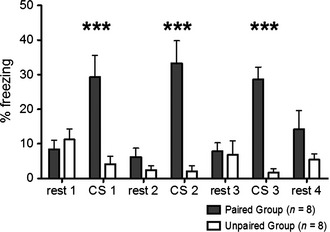
Behavioural assessment of the cued fear conditioning paradigm. % time freezing (± SEM) during retrieval, 24 h after conditioning. The paired group (PG) mice (grey bars) spent significantly longer freezing in response to all three conditioned stimulus (CS) presentations than the unpaired group (UG) mice (white bars; ***RM anova;* P *< 0.0001).

During the retrieval test, 24 h after fear conditioning, the PG spent significantly longer freezing during all three CS presentations than the UG [28% increase; 95% CI (18, 38); RM anova: group*CS *F*_1,14_ = 37.8, *P *< 0.0001; Fig. 4], confirming that the PG had learned to associate the CS with foot shock, while the UG had not.

### Brain activation in response to the CS in the PG mice

**Figure 5:**
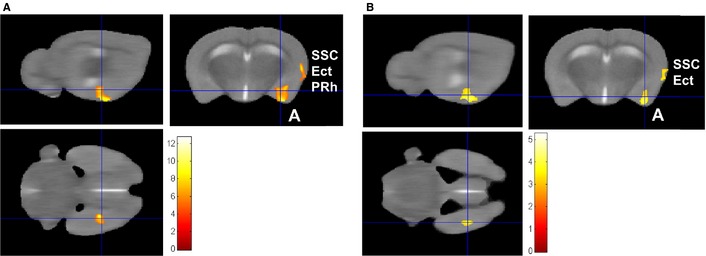
Statistical parametric maps (SPMs) showing brain activation [blood oxygen level‐dependent (BOLD signal] in response to the conditioned stimulus (CS). (A) SPM of paired group mice (PG,* n* = 8) showing a significant cluster of activation in the left amygdala region (denoted by A, and the cross‐hair; voxel‐wise *P*_uncorrected_ **< 0.001; cluster *P*_FWE_ = 0.013, coordinates 24, −18, −49). A cluster extending over the ectorhinal (Ect), perirhinal (PRh) and secondary somatosensory cortex (SSC) is also shown. (B) SPM of activation in response to CS that was greater in the PG than in the unpaired group (UG,* n* = 7), showing significant cluster in the left amygdala region [small volume correction (SVC) with left amygdala mask; cluster *P*_FWE_ = 0.031, coordinates 29, −16, −56]. Voxel‐wise threshold *P*_uncorrected_ **< 0.005 with a cluster extent threshold of 80 voxels for illustrative purposes, analysis conducted with voxel‐wise threshold *P*_uncorrected_ **< 0.001. Sagittal, coronal and axial views of the same cluster are presented, SPMs are presented overlaid on the average structural image (*n* = 15) and the right side of the image is the left side of the brain. Scale bar represents *T*‐score.

Within‐group analysis of brain activation in response to the CS compared with baseline in the PG mice revealed activation of the left amygdala region (*P*_FWE_ = 0.013, cluster extent = 751 voxels, peak *T* = 10.0, coordinates 24, −18, −49; Table 2; Fig. 5A). There was a trend for a cluster of activation that extended over the left ectorhinal cortex (Ect), perirhinal cortex (PRh) and the secondary somatosensory cortex (SSC; *P*_FWE_ = 0.068, cluster extent = 473 voxels, peak *T* = 12.7, coordinates 43, −14, −33; Table 2). Parametric modulation, used to model a linear change from first CS to the third CS, revealed no significant clusters of reduced or increased activation across the three CS presentations in the PG mice.

**Table 2:** Clusters of brain activation (BOLD response) in the PG (*n* = 8) and UG (*n* = 6)

Cluster statistic *P*_FWE corrected_	Cluster extent	Peak *T‐*score in cluster	Coordinates of peak voxel	Region
PG within group (CS > baseline), uncorrected threshold *P *< 0.001				
Whole‐brain analysis				
*P* = 0.013	751	10.0	24, −18, −49	Left amygdala
*P* = 0.068	473	12.7	43, −14, −33	Left PRh, SSC, Ect
PG > UG (CS > baseline), uncorrected threshold *P *< 0.001				
SVC using left amygdala mask				
*P* = 0.031	48	4.3	29, −16, −56	Left amygdala
UG within group (CS > baseline), uncorrected threshold *P *< 0.001				
Whole‐brain analysis				
*P *< 0.001	1197	15.5	20, 10, −44	Left NAcc Left LAcbSH
*P* = 0.059	289	16.4	−14, 4, −43	Right NAcc
UG > PG (CS1 > CS3), uncorrected threshold *P *< 0.001				
SVC using left amygdala mask				
*P* = 0.068	11	4.3	25, −23, −51	Left PMCo

*T* = peak voxel *t*‐statistic. CS, conditioned stimulus; Ect, ectorhinal cortex; LAcbSH, lateral nucleus accumbens shell; NAcc, nucleus accumbens core; PG, paired group; PMCo, posteromedial cortical amygdala nucleus; PRh, perirhinal cortex; SSC, secondary somatosensory cortex; SVC, small volume correction; UG, unpaired group.

Between‐group analysis (PG > UG) of brain activation, in response to the CS compared with baseline, revealed activation of the left amygdala region (voxel‐wise threshold *P*_uncorrected_ **< 0.001 with SVC *P*_FWE_ = 0.031, cluster extent = 48 voxels, peak *T* = 4.3, coordinates 29, −16, −56; Fig. 5B; Table 2). Parametric modulation (CS1 > CS3) revealed no significant increases or decreases in activation across the three CSs that was greater in the PG than in the UG.

Unthresholded images, which correspond to the raw effect size for each voxel and thus show all regions (uncorrected for multiple comparisons) for which there was some activation, show that both left and right amygdala, hypothalamus and granular insular regions were activated to a greater degree in the PG than the UG in response to the CS (Fig. S1). Similarly, unthresholded images of the pooled response of the PG and UG to the CS reveal activation in the primary visual cortex (Fig. S2).

### Brain activation in response to the CS in the UG mice

**Figure 6:**
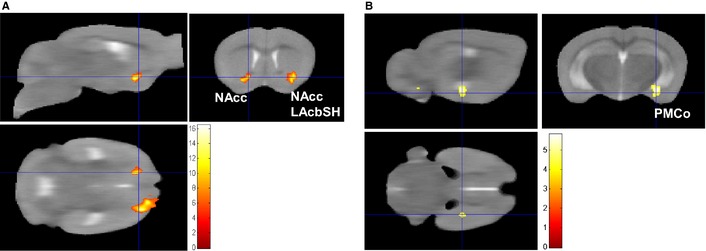
Statistical parametric map (SPM) of brain activation [blood oxygen level‐dependent (BOLD) signal] in response to the conditioned stimulus (CS) in the unpaired group (UG) mice. (A) A significant cluster of activation that extended over the left nucleus accumbens core (NAcc) and the left lateral nucleus accumbens shell (LAcbSH) was seen in response to the CS (voxel‐wise *P*_uncorrected_ **< 0.001; *P*_FWE_ **< 0.0001, cluster extent = 1197 voxels, peak *T* = 15.5, coordinates 20, 10, −44; cluster extent threshold 150 voxels for illustration purposes). The cluster in the right NAcc did not quite reach statistical significance (position of cross‐hair; voxel‐wise *P*_uncorrected_ **< 0.001; *P*_FWE_ = 0.059, cluster extent = 289 voxels, peak *T* = 16.43, coordinates −14, 4, −43). (B) The parametric modulation (CS1 > CS3) for the UG > paired group (PG) contrast revealed a cluster in the posteromedial cortical amygdala nucleus (PMCo) that reached a trend for significance [*P*_uncorrected_ **< 0.001; small volume correction (SVC) *P*_FWE_ = 0.068, cluster extent = 11 voxels, peak *T* = 4.3, coordinates 25, −23, −51]. Sagittal, coronal and axial views of the same clusters are presented, SPMs are presented overlaid on the average structural image (*n* = 15) and the right side of the image is the left side of the brain. Scale bar represents *T*‐score.

Within‐group analysis of brain activation in response to the CS compared with the baseline in the UG (*n* = 7) revealed a significant cluster of activation that extended over the left nucleus accumbens core (NAcc) and into the left lateral nucleus accumbens shell (LAcbSH; *P*_FWE_ **< 0.0001, cluster extent = 1197 voxels, peak *T* = 15.5, coordinates 20, 10, −44; Fig. 6; Table 2). There was a trend for a cluster of activation in the right NAcc (*P*_FWE_ = 0.059, cluster extent = 289 voxels, peak *T* = 16.43, coordinates −14, 4, −43; Fig. 6; Table 2). Between‐group analysis (UG > PG) of brain activation in response to the CS compared with baseline revealed the same cluster in the left NAcc, but it did not reach statistical significance when corrected for multiple comparisons across the brain with a voxel‐wise threshold *P*_uncorrected_ **< 0.001.

Parametric modulation (CS1 > CS3) revealed that there were no significant clusters of reduced or increased activation across the three CS presentations in the UG mice. However, parametric modulation using the contrast UG > PG revealed a cluster of activation in the left posteromedial cortical amygdala nucleus (PMCo); however, this cluster only reached a trend after SVC for the left amygdala (voxel‐wise threshold *P*_uncorrected_ **< 0.001; SVC *P*_FWE_ = 0.068, cluster extent = 11 voxels, peak *T* = 4.3, coordinates 25, −23, −51; Fig. 6B; Table 2). This suggests that amygdala activation was possibly reduced to a greater extent in the UG than the PG across the three CS presentations.

### Region of interest analysis: parameter extracts from left and right amygdala

**Figure 7:**
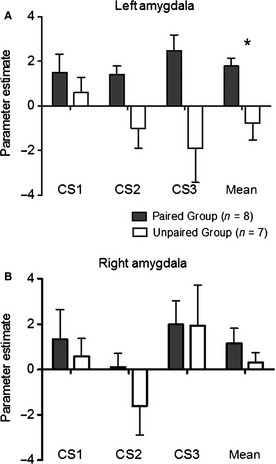
Parameter estimates extracted from left and right amygdala across the three conditioned stimulus (CS) presentations. Estimates of the relative response amplitude (± SEM) of neural activations elicited during the different conditions (CS1 > baseline, CS2 > baseline and CS3 > baseline) were extracted from an anatomically defined left amygdala for the paired group (PG;* n* = 8) and unpaired group (UG;* n* = 7). The zero level corresponds to the mean activation level during baseline periods (i.e. no CS exposure). (A) Left amygdala. (B) Right amygdala. The PG have, on average, a greater amygdala activation than the UG in response to the CS (*RM anova; main effect of group *P* = 0.03). The effect of group tended to depend on hemisphere (RM anova: group by hemisphere interaction: *F*_1,13_ = 4.0, *P* = 0.0674).

To further explore the amygdala response in the left and right hemispheres across the three CS presentations, the mean parameter estimates were extracted from an anatomically defined left and right amygdala mask for each mouse. The parameter estimates were significantly higher in the PG than the UG (Fig. 7; RM anova: main effect of group: *F*_1,13_ = 5.9, *P* = 0.0299), supporting greater amygdala activation in the PG. And there was a tendency for the effect of group to depend on the hemisphere (RM anova: group by hemisphere interaction: *F*_1,13_ = 4.0, *P* = 0.0674), which was due to a greater left response in the PG than the UG. However, strong support for lateralisation of amygdala activation was not found, as the left amygdala was not significantly more active than the right amygdala in the PG (paired *t*‐test: *t*_7_ = 1.5, *P* = 0.16). Finally, as found with the parametric modulation analysis, only weak support for a decrease in amygdala activation across the three CS presentations in the UG was found, and no evidence of extinction of amygdala activation in the PG (Fig. 7A).

## Discussion

Here the first study to use fMRI to investigate global network activation in awake mice that were processing a learned emotional stimulus was reported. Following a 12‐day acclimation protocol, which reduced movement and stress during scanning, activation of a well‐characterised fear network in response to the CS was successfully demonstrated. This significant methodological advancement demonstrates the feasibility of future fMRI studies in mice to investigate brain activation in genetic models of altered neural function, disease and aging.

Electrophysiological, lesion, fMRI and positron emission tomography studies confirm that the amygdala is key for the formation of fear‐related memories and emotional processing in humans and rodents (Morris *et al*., 1996; LaBar *et al*., 1998; Maren & Quirk, 2004; Phelps & LeDoux, 2005). Therefore, as expected, in response to the learned aversive stimulus a cluster of activation that incorporated both the basolateral complex (comprising the lateral, basolateral and basomedial nuclei) and the central nucleus of the amygdala was found. The basolateral complex receives convergent afferents from all sensory modalities and is instrumental in forming the CS–US association (reviewed in Maren, 2001). The central nucleus of the amygdala is the major output from the amygdala to downstream structures (e.g. brain stem), which mediate the behavioural and physiological response to the CS.

In addition to the amygdala, activation in the PRh, Ect and SSC was found in response to the CS. The PRh transmits visual (and auditory) sensory information about the CS to the basolateral complex of the amygdala, and lesions to the PRh block the expression of cue‐conditioned fear in rats (Campeau & Davis, 1995; Corodimas & Ledoux, 1995; Sacchetti *et al*., 1999). The SSC can project to the basolateral complex and is a component of a pain‐processing pathway that is recruited into the fear network in response to the CS (reviewed in McDonald, 1998).

No significant clusters of activation in visual‐processing regions in the PG or UG were detected; however, examination of the unthresholded map revealed that the primary visual cortex was indeed activated by the CS in the pooled response of the PG and UG (Fig. S2). Similarly, examination of the unthresholded map showed that the hypothalamus and granular insular regions were activated to a greater degree in the PG than the UG in response to the CS (Fig. S1), as found previously in the rat (Brydges *et al*., 2013). The unthresholded images correspond the raw effect size for each voxel and thus show all regions for which there was some activation (Jernigan *et al*., 2003), but because these clusters do not pass the strict FWE cluster correction used here, they cannot be reported as significant; however, the observed differences in this study and in Brydges *et al*. (2013) suggest that these regions do differ between PG and UG animals.

One rat study and a number of human fMRI studies have reported lateralisation of amygdala activation when processing emotional stimuli (LaBar *et al*., 1998; Phelps *et al*., 2001; Pine *et al*., 2001; Brydges *et al*., 2013), others find both sides are activated (Canli *et al*., 2000). Baas *et al*. (2004) conducted a meta‐analysis and concluded that the left amygdala is more often activated than the right in human fMRI studies of emotional processing. However, reports of hemispheric asymmetries should be interpreted with caution unless the hemisphere‐by‐task interaction is explicitly tested. In keeping with Baas *et al*. (2004) conclusion, our activation maps suggested unilateral amygdala activation, but a formal test of the hemisphere‐by‐task interaction on the extracted parameter estimates did not support this. However, the extracted parameter estimates were averaged across the whole amygdala complex, which may have precluded the detection of sub‐nuclei within the amygdala that have different processing roles within the left and right hemispheres, or if sub‐nuclei undergo different rates of extinction over repeated CS presentations. It is not possible to accurately distinguish between sub‐nuclei of the amygdala in this experiment because when preprocessing the data a spatial smoothing kernel was used that was twice the voxel size (Mikl *et al*., 2008). This served to increase statistical power by increasing the SNR at the subject level and reduce the impact of functional differences at the group level, but this level of smoothing comes at the cost of spatial precision. So although the well‐characterised neural pathway that mediates fear cognition was detected, it was not possible to accurately pinpoint peaks of activation within the clusters that were detected, for example, within sub‐nuclei (Mikl *et al*., 2008).

Brydges *et al*. (2013) report unilateral activation in the right amygdala in response to the CS; to explain this apparent discrepancy with the current data, it is highlighted that Brydges *et al*. (2013) did not formally test for lateralisation and it is possible that both sides were activated but below the statistical threshold (as in the current experiment).

To ensure the brain activation that was detected in response to the CS was specific to processing the learned fear, a control group of mice was included in which the cue was explicitly unpaired with the foot shock (UG mice). As found previously, the UG mice did not show any signs of fear to the CS in the behavioural retrieval experiment (Laxmi *et al*., 2003; Brydges *et al*., 2013). Furthermore, fear circuitry activation to the CS in UG animals in the fMRI experiment was not detected (Brydges *et al*., 2013). However, a significant cluster that encompassed the NAcc and the LAcbSH in the UG mice in response to the CS was detected. The NAcc is typically involved in processing motivational and reward‐related aspects of behaviour along with the ventral striatum, thalamus, orbitofrontal cortex and the caudate putamen (Liu *et al*., 2011). It is cautiously considered that the CS may have signified a safety signal to the UG mice, as previous studies have demonstrated that explicit unpairing of foot shock with a CS results in reduced fear‐related behaviour (e.g. increased exploration/reduced freezing) in response to the CS in the context in which the mice received the foot shock and reduced instinctive fear in an aversive novel environment (Rogan *et al*., 2005). Further, UG mice would actively seek to experience the CS that signalled safety and they also exhibited increased CS‐evoked electrophysiological field potentials in the caudate putamen (CS‐evoked responses were not investigated in the NAcc; Rogan *et al*., 2005). Therefore, it is tempting to speculate that the CS was a safety signal for the UG mice and that this was in some way ‘rewarding’.

While the NAcc has been assigned a prominent role in the processing of positive emotions, the NAcc has also been shown to play a role in processing expectancies (Pezze & Feldon, 2004), contingency awareness (Klucken *et al*., 2009) and animal studies reveal a role for the NAcc in fear conditioning (Pezze & Feldon, 2004; Iordanova *et al*., 2006). Moreover, the NAcc activation in the current study was not significantly greater in the UG than in the PG, raising the possibility that in this experiment the NAcc was involved in processing learned contingencies and processing the anticipation of a negative or positive outcome in both the PG and UG, respectively. A plausible scenario is that in the PG mice, NAcc activation is accompanied by amygdala activation, which coordinates the fear network response to the CS and means that these mice interpret the CS as unpleasant; whereas in the UG mice, NAcc activation is accompanied by a decrease in amygdala activation (Fig. 7A), which may mean these mice interpret the CS as a possible safety signal.

One major concern is that mice are stressed during fMRI scanning and that this may cause excessive motion or prevent the mouse from completing a cognitive task. Previous studies have successfully completed fMRI in rats and pigeons using surgically implanted head posts, which significantly reduces motion during scanning (Tsurugizawa *et al*., 2012; De Groof *et al*., 2013). However, the acrylic used to secure the post to the skull can cause scanning artefacts, requires regular care and can damage the skull. An alternative approach is to use restraint to hold the animal in place coupled with acclimation to scanning noises to reduce stress and motion (King *et al*., 2005; Febo, 2011). This approach has recently been successfully employed to image learned behaviour in awake rats, with physiological parameters reported to be within the range for resting rats in the active phase (Brydges *et al*., 2013; Johnson *et al*., 2013). Similarly, the current study found physiological parameters, including heart and respiration rates and levels of the stress hormone corticosterone, were within the normal range for a mouse during its active diurnal phase.

To further guard against motion‐related artefacts, a fast spin echo pulse acquisition sequence was used, which is less susceptible to physiological motion (head and cerebrospinal fluid movement) than gradient echo planar imaging sequences (reviewed in Ferris *et al*., 2006; Febo, 2011). Additionally, while gradient echo planar imaging may achieve faster acquisition rates and whole‐brain coverage, this sequence can be prone to signal losses and geometric distortion in the amygdala, primarily caused by the ear canal air–tissue interface (discussed in Febo, 2011). A trade‐off is that fast spin echo sequences are less sensitive to magnetic field inhomogeneity (and hence the BOLD signal); however, a high magnetic field can overcome this somewhat.

The human HRF was used to model mouse fMRI data. There may be differences in the HRF characteristics between species; however, previous work supports a linear neural–haemodynamic coupling relationship in the awake rat (Martin *et al*., 2006). Moreover, in this experiment, a block design of 2 min duration was used, during which the signal plateaus for the majority of the block, which limits the impact of any HRF inconsistencies between species and between individual animals.

In summary, the first study to use fMRI to investigate brain activation in awake mice that were processing a learned fear or safety‐related stimulus was reported. This work opens the door to future preclinical fMRI studies in mice to investigate genetic manipulations on brain function in models of disease.

## Supporting information

Fig. S1. Unthresholded activation maps showing regions of activation in response to the CS that was greater in the paired group than in the unpaired group mice.
Fig. S2. Unthresholded activation maps showing activation pooled across the paired and unpaired group mice (*n* = 14), specifically to investigate visual activation in response to the CS.
