# Supplementary material for: Imaging learned fear circuitry in awake mice using fMRI
Source: Eur J Neurosci. 2015 Jun 6;42(5):2125–34. doi: 10.1111/ejn.12939 (PMC4744695; doi:10.1111/ejn.12939)
Supplement: Supplementary file 1 — Fig. S1. Unthresholded activation maps showing regions of activation in response to the CS that was greater in the paired group than in the unpaired group mice. Fig. S2. Unthresholded activation maps showing activation pooled across the paired and unpaired group mice (n = 14), specifically to investigate visual activation in response to the CS. [file EJN-42-2125-s001.docx]

*Supporting Information*


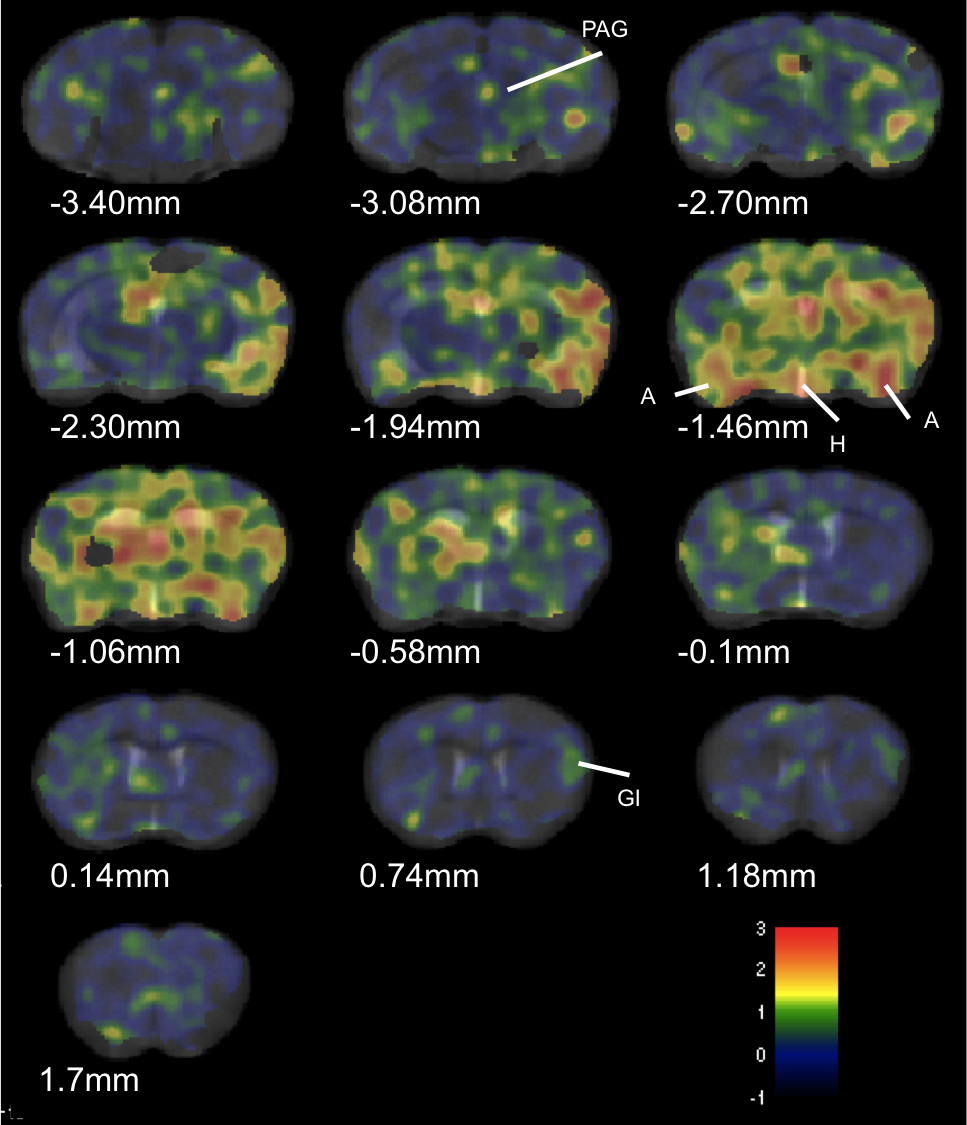


**Supporting Information; Figure S1. Unthresholded activation maps showing regions of activation in response to the CS that was greater in the paired group than in the unpaired group mice.** Activation is overlaid on the average structural template. Regions of interest are labeled: left and right amygdala (A), periaqueductal gray (PAG), hypothalamus (H) and the granular insular (GI) show activation in response to the CS. Scale bar represents the raw effect size (increase/decrease) in arbitrary units, the numbers represent approximate distance in mm from Bregma for each coronal slice based on the Franklin and Paxinos mouse brain atlas (Franklin & Paxinos, 2008). Right side of the image is the left side of the brain. Right side of the image is the left side of the brain.


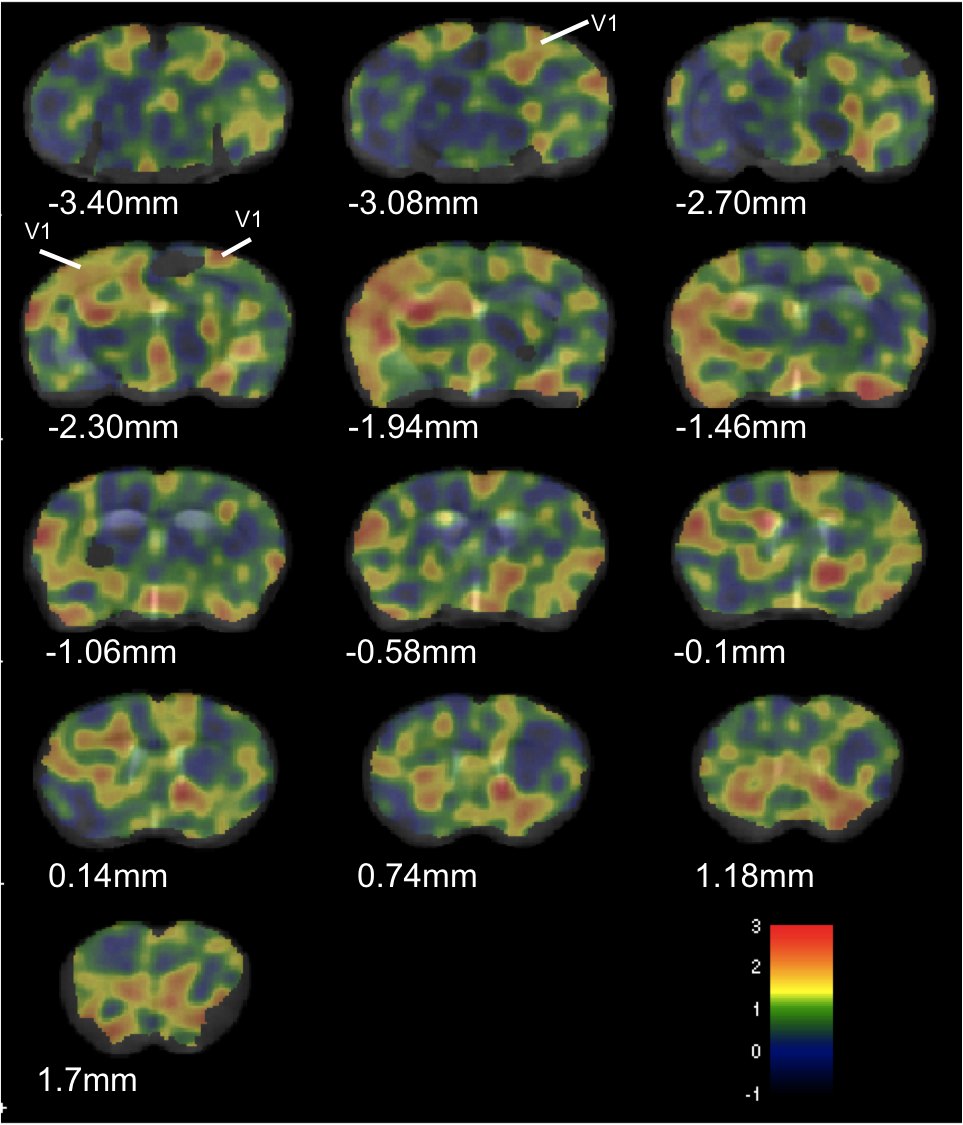


**Supporting Information; Figure S2. Unthresholded activation maps showing activation pooled across the paired and unpaired group mice (n = 14), specifically to investigate visual activation in response to the CS.** Activation is overlaid on the average structural template. The primary visual cortex (V1) is activated in response to the CS. Scale bar represents the raw effect size (increase/decrease) in arbitrary units, the numbers represent approximate distance in mm from Bregma for each coronal slice based on the Franklin and Paxinos mouse brain atlas (Franklin & Paxinos, 2008).
